# Feasibility and acceptability of expressive writing with postpartum women: a randomised controlled trial

**DOI:** 10.1186/s12884-018-1703-7

**Published:** 2018-03-27

**Authors:** Rosalind Crawley, Susan Ayers, Susan Button, Alexandra Thornton, Andy P. Field, Suzanne Lee, Andrew Eagle, Robert Bradley, Donna Moore, Gill Gyte, Helen Smith

**Affiliations:** 1 School of Psychology, University of Sunderland, Chester Road, Sunderland, SR2 7PT UK; 2 Centre for Maternal and Child Health Research, City, University of London, London, EC1V 0HB UK; 3 Department of Adult Nursing and Paramedic Science, University of Greenwich, London, SE9 2UG UK; 4 School of Psychology, University of Sussex, Brighton, BN1 9QH UK; 5 Central and North West London NHS Foundation Trust, London, W10 6DZ UK; 6 Brighton and Sussex University Hospital NHS Trust, Eastern Road, Brighton, BN2 5BE UK; 7 National Childbirth Trust, 30 Euston Square, London, NW1 2FB UK; 8 Division of Public Health and Primary Care, Brighton and Sussex Medical School, Brighton, BN1 9PH UK; 9 Lee Kong Chian School of Medicine Nanyang Technological University, Singapore, 308232 Singapore

**Keywords:** Feasibility, Acceptability, Expressive writing, Postpartum, Postnatal, Maternal

## Abstract

**Background:**

Pregnancy, birth and adjusting to a new baby is a potentially stressful time that can negatively affect women’s mental and physical health. Expressive writing, where people write about a stressful event for at least 15 min on three consecutive days, has been associated with improved health in some groups but it is not clear whether it is feasible and acceptable for use with postpartum women. This study therefore examined the feasibility and acceptability of expressive writing for postpartum women as part of a randomised controlled trial (RCT).

**Methods:**

The Health After Birth Trial (HABiT) was an RCT evaluating expressive writing for postpartum women which included measures of feasibility and acceptability. At 6 to 12 weeks after birth 854 women were randomised to expressive writing, a control writing task or normal care, and outcome measures of health were measured at baseline, one month later and six months later. Feasibility was measured by recruitment, attrition, and adherence to the intervention. Quantitative and qualitative measures of acceptability of the materials and the task were completed six months after the intervention.

**Results:**

Recruitment was low (10.7% of those invited to participate) and the recruited sample was from a restricted sociodemographic range. Attrition was high, increased as the study progressed (35.8% at baseline, 57.5% at one month, and 68.1% at six months) and was higher in the writing groups than in the normal care group. Women complied with instructions to write expressively or not, but adherence to the instruction to write for 15 min per day for three days was low (Expressive writing: 29.3%; Control writing: 23.5%). Acceptability measures showed that women who wrote expressively rated the materials/task both more positively and more negatively than those in the control writing group, and qualitative comments revealed that women enjoyed the writing and/or found it helpful even when it was upsetting.

**Conclusions:**

The feasibility of offering expressive writing as a universal self-help intervention to all postpartum women 6 to 12 weeks after birth in the HABiT trial was low, but the expressive writing intervention was acceptable to the majority of women who completed it.

**Trial registration:**

ISRCTN58399513, 10/09/2013.

## Background

Approximately 136 million women every year give birth [1] and of these, around 700,000 births take place in England and Wales [2]. For the majority of women, the experience of pregnancy and birth is positive, but for some, the challenge of adjusting to the physical and emotional changes that accompany childbirth is more difficult. For these women, the first weeks and months after birth are associated with physical recovery and a greater risk of experiencing psychological distress. Mental health challenges such as postpartum depression, anxiety and post-traumatic stress can have a devastating and enduring effect upon women and their families [3, 4]. The importance of early intervention and treatment for these women is emphasised in clinical guidelines but the lack of an evidence-base is also highlighted [5].

One intervention that may improve physical and psychological health is expressive writing [6]. Expressive writing interventions typically ask people to write their deepest thoughts and feelings about a stressful or upsetting event for at least 15 min every day for three days [7, 8].

The literature regarding the effect of expressive writing on psychological and physical health is somewhat mixed. Some meta-analyses suggest it is associated with small but consistent improvements in psychological and physical health [9–12]. However, others have concluded that while those who write often feel it is beneficial, the current evidence has not clearly demonstrated its effectiveness [13, 14]. Nevertheless, they acknowledge that there is a possibility that it may be beneficial for some health outcomes in particular groups of people in certain contexts, although these are still to be specified [14].

So far, only a few studies have specifically examined the effectiveness of expressive writing for postpartum women, but the evidence is encouraging. Results suggest that expressive writing is associated with improved psychological health after birth. However, most studies are restricted in some way, which limits how far the results can be generalised to postpartum women in general and to a range of health outcomes including physical health. Studies so far have either used expressive writing with mothers whose babies were in a Neonatal Intensive Care Unit (NICU) [15, 16], or they have primarily examined post-traumatic stress symptoms in mothers who were asked to write specifically about labour and delivery in the first week postpartum [17–20]. Some of these studies also used a variation on the expressive writing paradigm [15, 20]. Further evidence on the effectiveness of expressive writing for postpartum women is therefore needed [21].

The Health after Birth Trial (HABiT) was a large randomised controlled trial (RCT) carried out from 2013 to 2015 in England with the main aim of evaluating the effectiveness of expressive writing for postpartum physical and psychological health. At 6 to 12 weeks after birth, 854 women were randomised to take part in an expressive writing task, control writing task, or standard postpartum care. Women in the expressive writing condition were asked to write about a stressful event, which could be related to their pregnancy, birth, baby or something else going on in their life. Women in the control writing task were asked to write about a familiar room as objectively and dispassionately as they could. The results of HABiT showed that expressive writing had no effect on health outcomes. There were no differences between expressive writing, control writing and normal care groups on measures of physical health, anxiety, depression, mood or quality of life one and six months later [22].

The HABiT results are inconsistent with previous research in obstetric samples where all the published studies to date have found some positive benefit [15–20]. There are a number of possible reasons for this inconsistency; studies differed by country, writing instructions, when the intervention was offered, sampling, and outcome measures.

Cultural differences are possible since the HABiT study was conducted in England, whereas previous studies were conducted in Italy [17–20], Switzerland [16] and the USA [15]. What women were asked to write about and for how long also varied across studies. In some studies, women were asked to write about birth [17–20]. In others, women wrote about their most upsetting experience in NICU [15] or their most traumatic experience relating to the birth and hospitalisation of their preterm infant [16]. In HABiT, by contrast, women could write about anything stressful, and this could be associated with pregnancy, birth, the baby or something else. In the HABiT trial, women wrote for 15 min three times within one week. In other studies they wrote for 30 min for four days in a row [15], or 15 min on three consecutive days [16], or 15–20 min twice in one day [17], or only once, for 10–15 min and 20 min respectively [19, 20].

Timing of when the intervention is offered is likely to be particularly important, as the demands of caring for a new baby might make it difficult for women to find the time to write regularly without distractions, especially in the early postpartum period. In the HABiT trial, mothers were invited to write 6 to 12 weeks after birth. In previous studies, mothers wrote in the first week after birth [17–20], 2–14 months after their infant had been in NICU [15], and when their preterm infants were three months by corrected age [16]. Sampling also differed in that some previous studies specifically sampled high-risk women with babies that had been in NICU [15, 16], whereas HABiT used systematic sampling of all mothers to try to ensure a representative sample. Similarly, different outcome measures were used at different time points across studies.

Some of the inconsistent findings of previous research may be explained by the acceptability and feasibility of expressive writing in different contexts. The unique demands of the early postpartum period when women are adjusting to motherhood and coping with a new baby means expressive writing may not be feasible and/or acceptable for them to use at this time. If expressive writing is not feasible for women at this time in their lives and/or not acceptable to them then it is unlikely to be effective. This paper therefore aimed to evaluate (i) the feasibility and uptake of expressive writing and (ii) the acceptability of expressive writing to postpartum women in a non-selected, non-targeted population. In doing so, it can inform future research and clinical use of expressive writing with postpartum women.

## Methods

### Participants and procedure

HABiT was a parallel randomised controlled trial comparing expressive writing with a control writing task and normal care [22] which included measures of feasibility and acceptability. Women were recruited from 14 National Health Service (NHS) hospitals in England between November 2013 and December 2014. To be eligible to participate, women had to be at least 18 years old and to have given birth to a live infant at 26 weeks or more gestation. Women were excluded from the study if their baby was stillborn or died prior to discharge from hospital. Lived experience of mental health difficulties had no impact on eligibility.

All eligible women (*N* = 7986) in the 14 NHS hospitals were invited to take part approximately four to six weeks after birth. Staff in the hospitals put flyers in discharge packs and sent eligible women a letter inviting them to participate in the study, along with a participant information leaflet, consent form and reply-paid envelope. Women who were willing to take part were asked to provide their contact details, complete the consent form and state whether they wished to participate by post or by internet. Women who did not want to take part could respond with their reasons if they so wished.

**Fig. 1:**
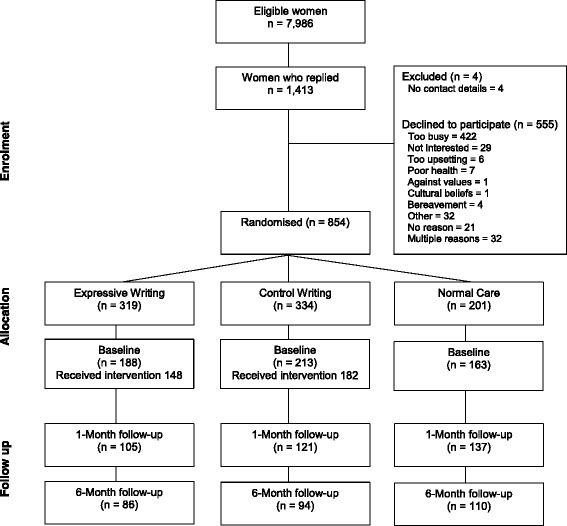
Sampling and attrition

Recruitment, allocation and sample attrition are shown in Fig. 1. A sample size calculation was carried out based on a study of the effect of expressive writing in a sample relevant to the current research, namely women undergoing infertility treatment [23]. Based on the results from a measure of stress, this showed that to detect a small effect in the primary outcome measures with a significance level of 0.05 and 80% power would require 122 women in each group, giving a maximum total sample of 366 across the three groups.

Of the women approached, 1413 replied and 854 consented and were randomised to expressive writing, control writing or normal care using a computerised random number generator. Following randomisation, women who elected to participate by post were sent the first workbook with a reply-paid envelope. If they did not return the workbook within ten days, they were sent reminders by post, email or text message. Women who wished to participate via the internet were registered on the study website from which they were sent an email or text message with their username and password. Women who did not log on and complete the workbook received reminder emails or text messages after seven days. The majority of women who took part chose to complete the study online (63.2%). Telephone follow-ups and regular newsletters were also used in an attempt to reduce attrition.

Women in all conditions completed baseline measures of mood, anxiety, depression, physical health, and quality of life. Women in the two writing conditions then completed the three-day writing task, followed by additional measures of mood, anxiety and depression. Women in the expressive writing group also rated their stress before and after each writing session. All women completed demographic measures of age, relationship status, education, ethnicity and employment. Follow-up measures of mood, anxiety, depression, physical health, and quality of life were collected one month and six months after baseline. Measures of acceptability were completed at six months. Women in the control writing or normal care groups were offered the opportunity to complete the expressive writing intervention at the end of the study if they wished to do so.

### Interventions

The expressive writing intervention was based upon Pennebaker’s expressive writing paradigm [7, 8]. Women were instructed to write for 15 min each day about a stressful event related to their pregnancy, birth, baby, or something else going on in their life. Women were asked to write about their “deepest thoughts and feelings” about this event. To avoid re-traumatising women who had suffered traumatic events, the instructions stated that if writing about this event felt too distressing or overwhelming women should pick a less stressful event. Women were asked to write on three consecutive days about the same event but, if they missed a day, to try to complete all three writing exercises within a week. The date and time they started and stopped writing was recorded automatically (online) or by self-report (postal). Before and after writing, women in the expressive writing condition were asked to rate how “upset or stressed” they were about the event they wrote about on a 10-point scale from 1 (*not at all*) to 10 (*extremely*). Finally, on the last day of writing women rated how distracted they had been whilst writing on a 10-point scale from 1 (*not at all*) to 10 (*extremely*).

The control writing task was matched to the expressive writing task for time and basic format. Women assigned to this condition were asked to write about a room for 15 min on three consecutive days. They were instructed to describe the same room on each day as objectively and factually as possible and not write about feelings, beliefs or opinions. They were asked to state which room they were writing about. A measure of how clearly they could visualise the room from 1 (*not at all*) to 10 (*extremely*) was taken before and after the writing task. The date and time they started and stopped writing and a measure of how distracted they had been whilst completing the writing tasks was also recorded.

### Measures

**Feasibility** was measured by examining recruitment, attrition, and adherence to the intervention. Measures of recruitment included: the proportion of potentially eligible women recruited; reasons for non-recruitment (*too busy, not interested, too upsetting, poor health, against personal values, adherence to cultural beliefs, bereavement or other*), and a measure of whether women were disappointed with the group to which they were randomised from 1 (*not at all*) to 10 (*extremely*). Attrition was examined by looking at how many women dropped out as the study progressed as a proportion of those who consented and were randomised (*n* = 854).

Feasibility in terms of adherence to the expressive writing task was evaluated by examining the total number of words written, the number of times each woman wrote (1–3), the total writing time (minutes), the number of days to complete the writing task (days), how distracted women were during writing (1–10), the proportion who wrote for at least 15 min on each of the three days, and the proportion who wrote for at least 15 min on all three days. Adherence to the instruction to write about thoughts and emotions in the expressive writing task was examined using Linguistic Inquiry Word Count (LIWC) [24] to compare the proportion of emotional words, cognitive words and perceptual words in the writing of the expressive writing and control writing groups. To investigate possible predictors of adherence, a number of theoretically motivated potential predictors (condition of writing task, baseline psychological symptoms, mental-health related quality of life, education level, complications at birth, and parity) were also examined.

**Acceptability** of the expressive writing intervention was assessed quantitatively and qualitatively six months after the intervention. A new rating measure of acceptability was developed for the quantitative measure of acceptability. Women were asked to rate the writing tasks and materials on 11 dimensions (*engaging, informative, interesting, bothering, clear, intrusive, upsetting, helpful, inviting, insightful, discouraging*) using a 10-point scale from 1 (*not at all*) to 10 (*extremely*). Factor analyses using principal axis factoring with direct oblimin rotation showed a clear two factor solution with a factor of positivity towards the task/materials (*engaging, informative, interesting, clear, helpful, inviting and insightful*) and a second factor of negativity to the task/materials (*discouraging, bothering, intrusive, upsetting*). Ratings of the task and materials was highly correlated (*r* = .773 and .827) suggesting women rated them very similarly. These were therefore combined into two overall subscales: positivity towards the materials/task; and negativity towards the materials/task. The correlation between positivity and negativity towards the materials/task was small (*r* = −.269) suggesting positivity and negativity are not measuring opposite aspects of the same underlying dimension. Internal reliability in our sample was high (positivity α = .96 (CI .95–.97); negativity α = .86 (CI .83–.89).

Qualitative measures of acceptability of the materials and tasks were obtained by inviting women to comment on the materials or task in an open text box. These were transcribed and exported into Excel where they were analysed using systematic thematic analysis to extract the main themes [25].

**Additional measures** were included to measure sociodemographic characteristics, basic obstetric details and information on complications during birth. Participants also provided information about any current or historic psychological difficulties and any medication being taken for a psychological condition.

**Trial outcome measures** of psychological health (mood [26], anxiety [27], depression [27]) physical health (physical symptoms [28], rating of overall health) and quality of life [29] have been reported elsewhere [22].

### Analysis

Analyses were conducted using R [30]. Comparisons of adherence measures were conducted across the two writing groups. Most of the adherence measures had outliers or were skewed, so groups were compared using robust methods based on 20% trimmed means and 2000 bootstrap samples: Wilcox’s yuenbt function in the WRS2 package [31]. Bayes factors were computed using the BayesFactor package [32].

## Results

### Sample characteristics

The final sample of women who provided some data (*n* = 564) was predominantly white European (94.7%), married or cohabiting (95.1%) and educated to degree level or higher (62.1%). Mean age of the participants was 32.77 years (*SD* 5.38; range 18 to 46 years). In comparison with United Kingdom (UK) norms, our sample was around two-and-a half years older than the average maternal age (30.4 years) [33], and a greater proportion were married (64.8%, compared with 47.5%). The majority of participants were employed (*n* = 327; 83%) and a large proportion of these worked in a professional occupation as defined by the standard UK classification system [34] (*n* = 132; 41.8%). There were no significant differences between intervention and control groups on sociodemographic or baseline measures. More detailed information on sample characteristics is given elsewhere [22].

### Feasibility of expressive writing for postpartum women

#### Recruitment and retention

An overview of recruitment, sampling and retention is shown in Fig. 1. Recruitment to the study was low, with only 854 of the 7986 eligible women (10.7%) agreeing to take part and being randomised. Of these, many women dropped out before completing the first workbook (*n* = 306). Some women went on to complete measures in later workbooks (*n* = 16) and their baseline measures were imputed.

As a percentage of the 854 women randomised to one of the three groups, attrition was 35.8% at baseline (Expressive writing 42.9%; Control writing 38.1%, Normal care 20.8%). Some of the women who completed baseline measures (*n* = 58) dropped out before starting the intervention (10.6% of those completing baseline measures). Retention was 57.5% at the one month follow up (Expressive writing 67.1%; Control writing 63.7%, Normal care 32.2%) and 68.1% at the six months follow up (Expressive writing 75.9%; Control writing 74.2%, Normal care 46.0%). The final sample for analysis was 564 women (66.0% of those randomised).

A generalized linear mixed model was used in which status (retained vs dropped out) as a level 1 variable was nested within participants (level 2). Dropout was predicted from fixed effects of condition (expressive writing, control writing or normal care) and time (0, 1 and 6 months from baseline). Dropout significantly increased over time (χ^2^(1) = 302.95, *b* = − 0.46, *SE* = 0.03, *z* = − 13.84, *p* < .001) and differed by condition (χ^2^(2) = 78.92, *p* < .001), with less dropout in the normal care group (*b* = 3.21, *SE* = 0.42, *z* = 7.61, *p* < .001) but no significant difference between expressive writing and control writing groups (*b* = 0.38, *SE* = 0.32, *z* = 1.19, *p* = .233). There was no significant interaction between groups over time in dropout, χ^2^(2) = 2.00, *p* = .367.

The groups differed significantly in how disappointed they were with the group to which they were randomised (*F*(2, 252) = 3.43, *p* = .034). The control writing group was the most disappointed (*M* = 2.35, *SD* = 2.70, range 1–10) followed by the normal care group (*M* = 1.71, *SD* = 1.71, range 1–8), and the expressive writing group was the least disappointed (*M* = 1.57, *SD* = 1.41, range 1–7). Only the range of ratings for the control group included 10, indicating extreme disappointment.

#### Reasons why women declined to take part

Some of the women who declined to participate (*n* = 555) provided their reasons for not taking part. These are shown in Fig. 1. Of those who declined, by far the most frequent reason was being *too busy* (*n* = 422, 75.9%). Other reasons, in order of decreasing frequency, were multiple reasons (*n* = 32, 5.8%); *other* e.g., concerns about confidentiality, already involved in other studies (*n* = 32, 5.8%); or *not interested* (*n* = 29, 5.2%).

#### Adherence to the writing interventions

Table 1 shows how well women in the expressive and control writing conditions adhered to the tasks, and their use of emotion, cognitive and perceptual words in their writing.

**Table 1:** Feasibility and acceptability of writing tasks

	Expressive writing Mean (*SD*) *N* = 188	Control writing Mean (*SD*) *N* = 213	Test statistic	*p*	*BF*_*01*_
**Feasibility of expressive writing**					
Adherence to writing tasks					
Total word count	680.31 (547.16)	592.09 (438.64)	*Y*_*BT*_ = 1 [−61.3, 186.93]	.326	0.513 ± 0%
Number of times women wrote:			χ^2^(3) = 5.55	.136	0.086 ± 0%
0 times (*n*, %)	*n* = 40 (21.3%)	*n* = 31 (14.6%)			
1 time (*n*, %)					
2 times (*n*, %)	*n* = 31 (16.4%)	*n* = 38 (17.8%)			
3 times (*n*, %)					
	*n* = 15 (8.0%)	*n* = 29 (13.6%)			
	*n* = 102 (54.3%)	*n* = 115 (54.0%)			
Total writing time in minutes	34.40 (26.92)	32.16 (22.34)	*Y*_*BT*_ = 0.59 [−4.34, 8.05]	.553	0.165 ± 0%
Days to complete writing task	4.61 (11.88)	6.21 (18.42)	*Y*_*BT*_ = −0.81 [−0.8, 0.33]	.416	0.183 ± 0%
Distractibility rating (range 1–10)	4.56 (2.44)	5.21 (2.50)	*Y*_*BT*_ = −1.90 [−1.54, 0.03]	.059	0.961 ± .01%
Women who wrote for 15 mins:					
Day 1 (*n*, %)	*n* = 117 (62.2%)	*n* = 120 (56.3%)	χ^2^(1) = 1.21	.273	0.258 ± 0%
Day 2 (*n*, %)	*n* = 81 (43.1%)	*n* = 99 (46.5%)	χ^2^(1) = 0.34	.561	0.157 ± 0%
Day 3 (*n*, %)	*n* = 71 (37.8%)	*n* = 71 (33.3%)	χ^2^(1) = 0.68	.411	0.199 ± 0%
All three days (*n*, %)	*n =* 55 (29.3%)	*n* = 50 (23.5%)	χ^2^(1) = 1.44	.230	0.331 ± 0%
Language categories in writing:					
Emotion words	6.20 (1.57)	1.66 (2.07)	*Y*_*BT*_ = 31.12 [4.62, 5.25]	< .001	5.84 ± 0%
Cognitive processing words	19.11 (2.65)	11.30 (3.22)	*Y*_*BT*_ = 27.03 [7.7, 8.91]	< .001	1.32 ± 0%
Perceptual words	2.69 (1.09)	4.76 (1.74)	*Y*_*BT*_ = −11.38 [−2.36, −1.67]	< .001	5.16 ± 0%
**Acceptability of expressive writing**					
Positivity of writing task	0.31 (0.82)	−0.28 (1.06)	*Y*_*BT*_ = 3.25 [0.26, 0.1]	.002	159.11 ± 0%
Negativity of writing task	0.46 (1.22)	−0.21 (0.70)	*Y*_*BT*_ = 4.21 [0.26, 0.76]	< .001	742.40 ± 0%

Adherence to the full writing intervention (to write for 15 min on three days) was low with only 29.3% of women in the expressive writing group and 23.5% of women in the control writing group complying with these instructions. However, the adherence measures showed no significant differences between the two writing groups. There were no significant differences between them in the total number of words written, the number of times they wrote, the total writing time in minutes, the number of days taken to complete the writing tasks, how distracted they were during the task, the proportion who wrote for at least 15 min on each individual writing day, or the proportion who adhered to the full writing instructions to write for at least 15 min on all three days. Averaging across the two writing groups, the mean number of words written was approximately 650 words over approximately five days, and the average time spent writing was about 33 min, although the standard deviations suggest there was substantial variation in these measures (Table 1). Just over half of the women wrote three times, but only just over one quarter wrote for at least 15 min on three days. The mean distractibility rating was approximately 5 on a 10-point scale. Thematic analysis of the stressful events written about on day one by women in the expressive writing group showed the most common topic concerned the baby (e.g. baby’s health, breastfeeding, crying baby, sleep, baby’s gender).

Analysis of the word categories women used in their writing showed significant differences in the word categories used in the expected direction. Women in the expressive writing group used a greater number of emotional words and cognitive processing words and fewer perceptual words than the control writing participants (Table 1).

To investigate possible predictors of adherence, a number of potential theoretically motivated predictors were examined using a logistic regression model. None were significant. Adherence (or not) was not predicted by whether women were in the expressive writing condition rather than the control condition (*b* = − 0.15, *SE* = 0.29, *p* = .61), had higher depression and anxiety scores at baseline (*b* = 0.02, *SE* = 0.02, *p* = .45); had poorer mental health related quality of life at baseline (*b* = 0.01, *SE* = 0.03, *p* = .57); were highly educated (GCSE or lower vs. A-level, *b* = 0.43, *SE* = 0.49, *p* = .38, and vs. degree, *b* = − 0.21, *SE* = 0.45, *p* = .64;); had fewer complications at birth (no complications vs. maternal, *b* = − 0.29, *SE* = 0.36, *p* = .42, vs. neonatal, *b* = 0.12, *SE* = 0.41, *p* = .78, and vs. both, *b* = 0.37, *SE* = 0.46, *p* = .43); or had fewer children (*b* = − 0.24, *SE* = 0.20, *p* = .23).

#### Undertaking expressive writing by internet or workbook

Just under two-thirds of women randomised chose to participate online (*n* = 540; 63.2%) as opposed to the workbook (*n* = 314; 36.8%). Choice of internet or workbook did not differ significantly between groups at the point of randomisation (χ^2^(2) = 1.64, *p* = .439). However, for women who completed the study, there were significant differences between groups with women in the two writing groups being significantly more likely to use the internet (expressive writing 73.4%; control writing 72.8%) than women in the normal care group (61.3%) (χ^2^(2) = 7.55, *p* = .022).

#### Acceptability of expressive writing

Table 1 shows that women in the expressive writing group rated the materials and writing task as both significantly more positive and more negative than women in the control writing condition. Qualitative comments about the writing tasks were provided by 79 women (Expressive writing *n* = 42; Control writing *n* = 37). Thematic analysis of these comments is shown in Table 2 and highlights some of the positive and negative aspects of expressive writing and control writing. There were fewer comments expressing negative experiences of expressive writing than expressing either positive or mixed experiences. Many women reported that they found the expressive writing task to be a positive experience. Some said they had enjoyed the task, that it had helped them to reflect and express their feelings, and that they had found it cathartic, or it had brought them some closure. Others spoke about developing insight into their feelings, or gaining a different perspective on the distressing event. However, some women did not find the expressive writing task particularly helpful and a few reported that it made them feel upset or sad. Some commented that the task was boring and repetitive, or a chore, and that writing repeatedly about the same distressing event reinforced the negative views they were expressing. A number of women described their experience of expressive writing as mixed, saying that even though it was distressing, time consuming, or left them ruminating about the event, they had found the writing exercise helpful. Some women pointed out that they had been prepared to feel some upset and they did not consider this a problem. The format of the expressive writing task was mentioned by some women who said it did not suit their needs, for example, they found the first writing session helpful but not the subsequent sessions.

**Table 2:** Qualitative themes on positive and negative aspects of writing tasks

Theme	Illustrative quotes
Positive feelings and outcomes of expressive writing	*I have found this exercise really helpful. I haven’t expressed my feelings before in this way and has been quite therapeutic to have free reign on my emotions in relation to this topic.* (F310)
	*I found writing about this experiences very helpful - as I wrote it seemed to order my thoughts and by the third writing experience I could put into words why I was feeling so bad.* (G317)
	*I enjoyed taking part in the writing tasks I did, it helped to write it all down!* (E303)
	*I found doing this workbook really helpful. It’s made me see a bit clearer on what’s going on in my life and day to day. I feel more positive after opening up my feelings and emotions.*(J007)
	*I have found completing this quite liberating and it has brought about some closure to what happened to me.... I have found it useful to talk about what happened and get some clarity in my mind of how things happened.*(B420)
	*I’ve found this so cathartic…* (E662)
Negative feelings and outcomes of expressive writing	*Found writing about my worst experience very upsetting.* (E309)
	*I think deliberately focusing on a stressful thing for as long as 15 mins made me more stressed than if I’d been allowed to set out my thought on it, resolve them, and move on. So I don’t think this whole exercise has so far been very helpful to me.* (G178)
	*I found the writing tasks boring and repetitive. I hope they are helpful to others, but they certainly weren’t helpful to me, in fact they can reinforce negative feelings.* (A104)
Mixed feelings and experiences of expressive writing	*The writing task was by its nature v. personal hence “intrusive” is rated highly - for me it was difficult hence “upsetting” - but I don’t think either of these are a ‘problem’ - that is what I knew I had signed up for!* (A1083)
	*Although distressing it has helped, and since 1st booklet I’ve started a diary where I write how I feel. It helps to write!* (C659)
	*It has been quite helpful to write about some of the difficulties I have had, although I found some of the writing exercises a bit repetitive.* (H922)
	*It has helped but also made me think about the situation more than I was.* (G811)
	*Although it’s taken me ages to complete I’ve gained a lot from it.* (A139*)*
Positive feelings and perceived benefits of the control writing task	*Writing tasks really helped me focus and I sit and write now when/if I feel I’m struggling. It helps clear my head.* (E845)
	*I definitely felt calmer and more relaxed after completing each writing exercise. It allowed me time to focus on something that wasn’t on my mind almost like a distraction.* (H662)
	*The task helped me to focus and relax and feel a little more like my old ‘pre-baby’ self.* (H998)
	*I have enjoyed the writing part of the exercise and have felt different after, more relaxed.* (A067)
Negative feelings and perceived negatives of the control writing task	*I was rather disappointed to find myself in the non-intervention arm of this study. I was hoping to gain some therapy from taking part in the study as my son’s birth turned traumatic towards the end.* (A772)
	*I did find the writing about a room part quite difficult and not an interesting topic to write about.* (F250)
	*I found it quite tiresome and difficult to write ‘dispassionately’ for 15 mins.* (E905)
Mixed feelings and experiences of the control writing task	*Whilst writing in the workbook distracted for a short while, I did not find it useful in terms of ‘working through’ some of my feelings.* (A533)
	*While writing had a slightly calming effect, and has helped me to focus on something else, it has taken up time in my day where I should have rested. This has meant that my mood may have worsened due to feeling that I’ve not had any ‘down time’.* (E644)
Barriers to completing the writing tasks	*Quite frankly finding 15 min of predictable uninterrupted peace with a little baby is very difficult. He is currently sitting on my lap and screaming as I finish this off.* (A237)
	*Been hard to complete with two children to look after and a house to run.* (E372)
	*I did forget about the day three task with getting caught up with looking after the three children.* (B676)
	*I was unable to fulfil the (writing task) as it was largely a written exercise, and due to struggling with learning to be a new mother and feeling low much of the time…* (E041)
	*...the website crashes frequently and is very slow to load.* (A1117)

Comments about the control writing task were mixed. Some women reported that although they had experienced positive feelings or benefits, such as enjoyment or distraction, these were moderated by negative feelings such as boredom or taking time from more worthwhile activities, so their overall sense was that they were not experiencing a therapeutic gain for their efforts. Perceived benefits of the control writing task were focusing the mind, clearing the head, and providing a distraction from other demands in their lives. Some women reported that they enjoyed the task and felt calmer, more relaxed, and more like themselves after writing. Negative feelings included disappointment, frustration, boredom and annoyance about the control writing task. Some women stated how difficult they found it not being able to write about their feelings. Others expressed disappointment that they had been allocated to the control writing group and to what they saw as a pointless task, when the time could have been spent on other activities perceived as more important, such as relaxation, or spending time with their family.

A number of barriers to completing writing tasks were mentioned. Women most commonly mentioned lack of time as a barrier, particularly when they had other children to look after as well as their new baby. Others found that the task got more laborious each day and this was related to the time commitment involved, boredom, and how beneficial they personally found writing. Other cognitive and emotional factors were mentioned as barriers, such as the effort expended in learning how to be a mother, dealing with emotional difficulties, and remembering to write each day. In addition, some of those who completed the study online cited technical difficulties with the website.

## Discussion

The results showed that the feasibility of offering expressive writing as a universal self-help intervention to all postpartum women 6 to 12 weeks after birth in the HABiT trial was low. One aspect of this was recruitment, which was much lower than expected. Originally, it was anticipated that 2100 women from four NHS hospitals would need to be approached for the study to be adequately powered. In fact, nearly four times that number from 14 NHS hospitals had to be approached. Recruitment rates in other studies of expressive writing with postpartum women have varied a great deal, and in some studies rates were much higher, between 80 and 90% [16, 17, 20]. Multiple differences in methodologies make it difficult to account for these differences but possible reasons for higher rates include face-to-face recruitment used in other studies [16, 17, 20] and the timing of the invitation to write. Inviting women to take part in an expressive writing intervention only 6 to 12 weeks after birth may be too demanding. A similarly low level of recruitment to HABiT was reported in a study of expressive writing with a sample of women with postpartum PTSD [35]. While the mental health of the sample may have contributed to low recruitment, it may also have been because of the timing of the intervention, which started at six weeks postpartum. In HABiT the most common reason mothers gave for declining to take part was that women were too busy, supporting the suggestion that asking women to write in the very early postpartum period is not feasible. In another study, mothers were invited to write later in the postpartum period (when their preterm baby was three months by corrected age) and the majority of women (70.5%) found this timing acceptable [16]. A more thorough examination of the feasibility of the timing of the intervention prior to the HABiT trial would have been advisable.

Another feasibility issue was the restricted range of sociodemographic characteristics of the women who agreed to participate in HABiT. Although the characteristics of the women who declined to take part or dropped out before baseline were not available, it is clear that the remaining sample does not represent the general population. A high proportion were white European, well-educated and employed, many in professional occupations. Compared with UK norms, they were older and more likely to be married. Simply writing to mothers early in the postpartum period to offer expressive writing online or in a workbook is not likely to be successful in reaching women with a range of sociodemographic profiles. It may be more effective for a recommendation to be made in a consultation with a healthcare professional.

Attrition rates also indicated a problem with feasibility of the intervention. Attrition levels were high and increased as the study progressed, particularly in the two writing groups. RCTs examining women’s health frequently fail to achieve target sample sizes [36] and there is substantial variability in attrition rates in studies with postpartum women. Some report levels as high as in HABiT e.g., [37] while others report lower levels e.g., [38]. Attrition levels are also variable in studies that have focused specifically on postpartum writing interventions. There was very low or no attrition in some studies [15, 17, 20] but high attrition in others [16].

Although there is no agreed threshold for acceptable attrition rates, the levels in HABiT were well above the 20% that is generally considered to be a concern [39, 40]. Lack of time and being busy with a newborn baby may explain the high attrition rates and also why more women dropped out from the writing groups than from the normal care group. In the early postpartum period women are adjusting to motherhood and coping with a new baby so finding the time and effort needed to write three times for 15 min per day is likely to be difficult. For the many mothers who had other children to care for as well as their new baby, lack of time is likely to have been a particular issue. The challenges of this time are evident from the finding that the baby was the most common topic in the expressive writing narratives about what mothers were finding stressful. Furthermore, when asked to comment on how they found the writing tasks, many women reported that finding the time to write was a barrier.

Adherence measures showed good adherence to the writing task in terms of writing expressively or not. The two writing groups did not differ in how much they wrote, for how long, or how often, but differences in their word category use were consistent with their instructions to write expressively or descriptively. However, adherence to the instructions for the writing task (15 min of writing for three days) was low (under 30%), and lower than reported in some studies of expressive writing with other samples. For example, it was approximately 50% in one study [41] and 100% in others [15, 16]. However, in another study of expressive writing where mothers with postpartum PTSD were asked to write 6 to 12 weeks after the birth of their baby [35], adherence was even lower than in HABiT. While the mental health of the sample may have been partly responsible for this, it is likely that the timing of the intervention was also important. In the two studies where women were asked to write 6 to 12 weeks after the birth of their baby, adherence was low [22, 35], yet it was high in studies that asked women to write later in the postpartum period [15, 16].

Even though participants were aware that the intervention involved expressive writing, and ratings and qualitative comments showed that control writing participants were more disappointed with the group to which they were randomised, attrition was no higher and adherence was no lower in the control writing group compared with the expressive writing group. In a study of expressive writing for people with mood disorders, adherence was more likely in participants with lower levels of psychological distress and fewer physical health complaints [41]. By contrast, in HABiT, adherence to the intervention was just as likely if women had poorer mental health outcome scores at baseline, if they had experienced more complications at birth, or had more children.

Measures of acceptability showed some differences between the writing groups. Women in the expressive writing group rated the materials and writing task as both more positive and more negative than women in the control writing group. Nevertheless, the qualitative comments from women in the expressive writing group included more that were positive or mixed rather than negative. This suggests that the intervention was acceptable to most women who responded, and supports previous research suggesting that expressive writing was acceptable to mothers in the postpartum period [16]. Detailed comments from women who wrote expressively revealed that some enjoyed the task, and others found it helpful despite negative aspects of the experience, such as finding it upsetting to express their thoughts and emotions or finding the task boring and repetitive.

The reported benefits were consistent with explanations for the benefits of expressive writing [9], such as helping women gain insight and understanding, see a different perspective, achieve closure, or finding it cathartic to express their feelings. While some women in the control writing group also said they enjoyed writing, the reported benefits were different. Typically, they said it was calming and relaxing to focus on something other than their current concerns. The negative aspects of the task also differed in some ways. While women in both writing groups found the task boring and repetitive, women in the control writing group said it was pointless and disappointing to be in that group. When women in the two groups expressed feelings that were mixed, they differed in emphasis. Women given the control writing task were more likely to put the emphasis on the negative than the positive aspects of the experience (e.g., *While writing had a slightly calming effect, …. it has taken up time in my day where I should have rested*), while those who wrote expressively more often stressed positive aspects of the experience (e.g.*, Although distressing it has helped)*. Thus, the qualitative comments suggest that the expressive writing task was acceptable but finding the time to write was difficult. The barriers to writing mentioned by women in both writing groups often centred on the difficulty of finding time to write given the demands of adapting to motherhood and looking after a new baby.

A major strength of the HABiT study is that it is the first and rigorous evaluation of the feasibility and acceptability of expressive writing with postpartum women, based on a RCT. A large number of women completed the measures providing valuable data about the likelihood that expressive writing is feasible and acceptable as a universal intervention to improve postpartum health. It used a variety of dimensions and measures of feasibility and acceptability including a new rating measure of the acceptability of materials and tasks that could be used to elsewhere to assess the acceptability of writing or similar tasks for postpartum women.

There were some limitations of our study. One is not being able to compare the sociodemographic characteristics of the women who did not take part with those who did. Another limitation is that women were not asked about the acceptability of the expressive writing intervention until the follow up six months later. By this time, it might have been difficult for women to remember what they thought about the materials and the task they encountered six months before. Furthermore, the high rates of attrition by six months means that many women did not provide data on acceptability.

This study has a number of implications for research. It demonstrates the importance of examining feasibility and acceptability in studies in the postpartum period because of the particular demands on women at this time. It is clear that 6 to 12 weeks after birth is not a good time to recruit women to studies that demand time and focus, particularly if involvement is required over a period of time. It also shows the importance of using both qualitative and quantitative measures. It would have been difficult to interpret the finding that women rated the materials/task both positively and negatively without the comments women provided. In terms of research using expressive writing at a distance (online or by post) in the postpartum period, it is important to be aware of the large number of participants that need to be approached to achieve adequate power. In order to recruit and retain participants, it is suggested that the study should either be conducted later in the postpartum period or, if it is important to use expressive writing soon after birth, it is best to do so in hospital, where women have more support with the care of their baby and few other demands on their time. Alternatively, particularly for women who are discharged soon after birth, it is important to find other ways to provide women with support, for example, by engaging partners to create protected time for them to write.

The study also has implications for clinical practice. It suggests that expressive writing is acceptable to the women who do it, but that those who respond to the invitation will be a self-selected group with particular characteristics, so if it is to be offered as an intervention then it is not likely to be taken up universally. This information is useful for informing postpartum care in developed countries.

## Conclusions

Acceptability measures from the HABiT trial evaluating the effectiveness of expressive writing for postpartum physical and psychological health showed that the expressive writing intervention was acceptable to the majority of women who completed it. However, feasibility measures suggest that, when expressive writing is offered as a universal self-help intervention, it is not feasible for postpartum women 6 to 12 weeks after birth.

## Abbreviations

HABiT: Health after Birth Trial
LIWC: Linguistic Inquiry Word Count
NHS: National Health Service
NICU: Neonatal Intensive Care Unit
PTSD: Post-traumatic stress disorder
UK: United Kingdom
